# A mRNA-Responsive G-Quadruplex-Based Drug Release System

**DOI:** 10.3390/s150409388

**Published:** 2015-04-21

**Authors:** Hidenobu Yaku, Takashi Murashima, Daisuke Miyoshi, Naoki Sugimoto

**Affiliations:** 1 Advanced Research Division, Panasonic Corporation, 3-4 Hikaridai, Seika-cho, Soraku-gun, Kyoto 619-0237, Japan; E-Mail: yaku.hidenobu@jp.panasonic.com; 2 Faculty of Frontiers of Innovative Research in Science and Technology (FIRST), Konan University, 7-1-20 Minatojima-Minamimachi, Chuo-ku, Kobe 650-0047, Japan; E-Mails: murasima@konan-u.ac.jp (T.M.); sugimoto@konan-u.ac.jp (N.S.); 3 Frontier Institute for Biomolecular Engineering Research (FIBER), Konan University, 7-1-20 Minatojima-Minamimachi, Chuo-ku, Kobe 650-0047, Japan

**Keywords:** drug delivery carrier, G-quadruplex, anionic phthalocyanine, cancer, mRNA, telomerase

## Abstract

G-quadruplex-based drug delivery carriers (GDDCs) were designed to capture and release a telomerase inhibitor in response to a target mRNA. Hybridization between a loop on the GDDC structure and the mRNA should cause the G-quadruplex structure of the GDDC to unfold and release the bound inhibitor, anionic copper(II) phthalocyanine (CuAPC). As a proof of concept, GDDCs were designed with a 10-30-mer loop, which can hybridize with a target sequence in epidermal growth factor receptor (EGFR) mRNA. Structural analysis using circular dichroism (CD) spectroscopy showed that the GDDCs form a (3 + 1) type G-quadruplex structure in 100 mM KCl and 10 mM MgCl_2_ in the absence of the target RNA. Visible absorbance titration experiments showed that the GDDCs bind to CuAPC with *K*_a_ values of 1.5 × 10^5^ to 5.9 × 10^5^ M^−1^ (*K*_d_ values of 6.7 to 1.7 μM) at 25 °C, depending on the loop length. Fluorescence titration further showed that the G-quadruplex structure unfolds upon binding to the target RNA with *K*_a_ values above 1.0 × 10^8^ M^−1^ (*K*_d_ values below 0.01 μM) at 25 °C. These results suggest the carrier can sense and bind to the target RNA, which should result in release of the bound drug. Finally, visible absorbance titration experiments demonstrated that the GDDC release CuAPC in response to the target RNA.

## 1. Introduction

Drug delivery systems (DDSs) represent an important tool for preventing drug side effects [1]. DDSs are of particular interest for cancer therapy because most conventional chemotherapeutic anticancer drugs are distributed nonspecifically in the body and affect both normal and cancer cells. DDSs can be broadly categorized into two types: those that selectively deliver the drug to target cells, and those that stimulate the target cells to affect a therapeutic response. Many DDSs belonging to the first category have been approved for clinical use or are being evaluated in clinical trials [2,3,4,5]. In contrast, research into DDSs belonging to the second category is just beginning. There are many differences between the microenvironments of normal and cancer cells, including oxygenation, pH, metabolic state, and the amounts of mRNA. As our understanding of these differences increases, the development of stimulus-responsive DDSs should accordingly increase [6,7,8,9,10,11].

DNA is promising as a drug delivery carrier for stimulus-responsive DDSs because DNA changes conformation upon recognizing target nucleic acids in its environment [12,13]. In particular, the conformational change driven by hybridization reactions with the target nucleic acid is highly specific [12,13,14,15]. Various dynamic DNA devices utilizing hybridization reactions have been proposed. For example, it was reported that the conformational change of a thrombin-binding G-quadruplex to double-stranded DNA (dsDNA) via hybridization with the complementary DNA causes release of thrombin [16,17]. In addition, Bourdoncle *et al.* developed a G-quadruplex-based molecular beacon with a loop that is complementary to the target DNA [18]. These results indicate that it is possible to design functional G-quadruplexes for any desired target sequence. However, there has been no report of a G-quadruplex-based drug delivery carrier (GDDC) utilizing hybridization with target sequences to control the capture and release of a drug. 

**Figure 1:**
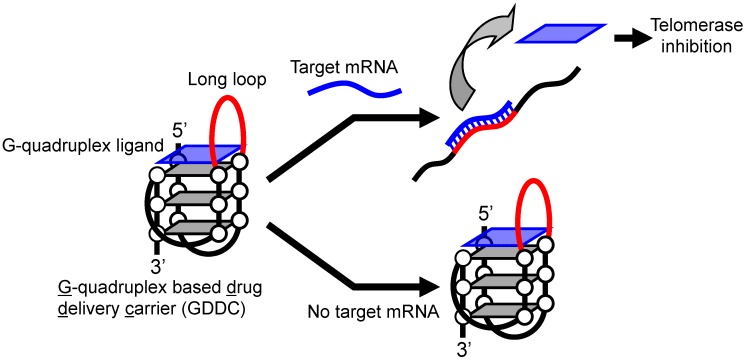
Principle behind DDS using a GDDC for cancer therapy. In the absence of a target mRNA in normal cells, the GDDC retains its G-quadruplex structure and thus a G-quadruplex ligand (a telomerase inhibitor) remains bound. In the presence of the target mRNA in cancer cells, the G-quadruplex structure of the GDDC unfolds, releasing the G-quadruplex ligand and inhibiting telomerase activity.

The GDDC should capture its drug cargo. Many π planar compounds are ligands for human telomeric G-quadruplex and bind [19,20,21,22,23,24,25,26]. Besides, such G-quadruplex ligands inhibit the activity of telomerase, which plays a critical role in the indefinite division and growth of cancer cells by elongating telomere DNA [19,20,21,22,23,24,25,26,27,28,29]. Thus, a GDDC that can release the G-quadruplex ligand in response to environmental factors specific to cancer cells should allow cancer therapy with higher drug efficiency and fewer side effects. Here, we developed a GDDC with a long loop which is complementary to the target mRNA (Figure 1). In the absence of the target mRNA, the GDDC maintains its G-quadruplex structure and can capture copper(II) anionic phthalocyanine (CuAPC), a highly specific G-quadruplex ligand and telomerase inhibitor [13,30,31,32]. CuAPC bound to GDDC cannot inhibit telomerase. However, high transcription of the target mRNA in cancer cells results in hybridization between the target mRNA and the long loop of the GDDC, causing unfolding of the G-quadruplex structure. The target mRNA-responsive unfolding of the GDDC releases CuAPC and telomerase is inhibited. As a proof of concept, we designed four GDDCs. The four GDDCs contain a 10–30 mer length loop targeting epidermal growth factor receptor (EGFR) mRNA, which is transcribed at high levels in cancer cells [33]. CD analysis, visible absorbance titration, and fluorescence titration experiments demonstrated that each G-quadruplex folds into a (3 + 1) type G-quadruplex structure [34,35,36] and can capture and release CuAPC in response to a target oligoRNA corresponding to EGFR mRNA.

## 2. Experimental

### 2.1. Materials and Reagents

All deoxyribooligonucleotides and ribooligonucleotides were high-performance liquid chromatography purification-grade and were purchased from Tsukuba Oligo Service Co., Ltd. (Ibaraki, Japan). A TRAPEZE telomerase detection kit was purchased from EMD Millipore Corporation (Billerica, MA, USA). HeLa cells included with the TRAPEZE telomerase detection kit were used. PCR was conducted using TaKaRa LA Taq HS polymerase provided with TaKaRa LA Taq Hot Start Version from Takara Bio Inc. (Shiga, Japan). CuAPC, 5,10,15,20-Tetra-(*N*-methyl-4-pyridyl)porphyrin (TMPyP4) [37], and Fe(III)-protoporphyrin IX (hemin) [38] were purchased from Sigma-Aldrich Japan K. K. (Tokyo, Japan), Dojindo Laboratories (Kumamoto, Japan), and Tokyo Chemical Industry Co., Ltd. (Tokyo, Japan), respectively, and were used without further purification.

### 2.2. Preparation of Cell Lysate

A pellet of 10^6^ Hela cells was suspended in 200 μL of cold CHAPS lysis buffer provided with the TRAPEZE telomerase detection kit. The cell lysate solution was divided into small volume aliquots and stored at −80 °C. Each aliquot was diluted in cold CHAPS lysis buffer as appropriate before use. 

### 2.3. G-quadruplex Structural Analysis

CD experiments for 20 μM a human telomeric oligo DNA (HteloDNA) and GDDC (GDDC-L10, L15, L20 or L30) in a buffer containing 50 mM MES-LiOH (pH 7.0), 100 mM KCl, and 10 mM MgCl_2_ were carried out using a J-820 spectropolarimeter (JASCO Co., Ltd., Hachioji, Japan) with a 0.1-cm path-length quartz cell at 25 °C. The CD spectrum was obtained by taking the average of three scans made at 0.5-nm intervals from 200 to 350 nm. Before measurement, the DNA sample was heated at 80 °C for 2 min for denaturation of the DNA structure, then gently cooled at 2 °C·min^−1^ to make the most stable DNA structure in the experimental condition. The most stable structure is required for quantitative analysis for thermal stability of G-quadruplex and for Cu-APC binding with DNA structures.

UV melting curves of G-quadruplexes formed by HteloDNA and each GDDC in the same buffer containing 50 mM MES-LiOH (pH 7.0), 100 mM KCl, and 10 mM MgCl_2_ were measured at 295 nm. A spectrophotometer (UV-1700, Shimadzu Co., Ltd., Kyoto, Japan) was used with a 1.0-cm pathlength quartz cell. Before measurement, the DNA samples were heated at 90 °C for 1 min, then gently cooled to 0 °C at 0.5 °C·min^−1^. The DNA samples were then heated from 0 to 90 °C at 0.5 °C·min^−1^.

### 2.4. UV-Vis Spectral Analysis

CuAPC (2.5 μM), TMPyP4 (1.0 µM), or hemin (12.5 µM) were mixed with various concentrations of G-quadruplex (HteloDNA, GDDC-L10, L15, L20 or L30) and a target oligo RNA corresponding to EGFR mRNA (rEGFR), and UV-Vis spectra were recorded at 25 °C. A Shimadzu UV-1700 spectrophotometer with a 1.0-cm path length quartz cell was used. The measurements for 1.0 µM TMPyP4 and 2.5 µM CuAPC were carried out in a buffered solution comprising 50 mM MES-LiOH (pH 7.0), 100 mM KCl, and 10 mM MgCl_2_. The measurements for 12.5 µM hemin were carried out in a buffer comprising 50 mM MES-LiOH (pH 7.0), 100 mM KCl, 10 mM MgCl_2_, and 2 mM NaOH. Each sample was heated to 80 °C for 2 min and gently cooled to 20 °C at a rate of 2 °C·min^−1^ before measurements.

### 2.5. Determination of the Association Constant

The fractional degree (ν) of saturation of each ligand-binding site on DNA can be expressed by the following equation based on a model assuming one binding site [39]:

(1) $$\nu =\left\{K_{d}+\left[\mathrm{ligand}\right]+\left[\mathrm{DNA}\right]-\sqrt{{\left(K_{d}+\left[\mathrm{ligand}\right]+\left[\mathrm{DNA}\right]\right)}^{2}-4K_{d}\left[\mathrm{ligand}\right]\left[\mathrm{DNA}\right]}\right\}/2\left[\mathrm{ligand}\right]$$

where *K*_d_ is the dissociation constant, [DNA] is the concentration of DNA, and [ligand] is the concentration of the ligand. Equation (1) can be transformed into Equation (2). The association constant (*K*_a_) for each DNA and its ligand was determined with Equation (2):

(2) $$\theta =a\left\{K_{a}\left[\mathrm{ligand}\right]+K_{a}\left[\mathrm{DNA}\right]+1-\sqrt{{\left(K_{a}\left[\mathrm{ligand}\right]+K_{a}\left[\mathrm{DNA}\right]+1\right)}^{2}-4{K_{a}}^{2}\left[\mathrm{ligand}\right]\left[\mathrm{DNA}\right]}\right\}/2K_{a}\left[\mathrm{ligand}\right]+b$$

where *θ* is the absorbance value, *K*_a_ is the apparent association constant of DNA/ligand binding, *a* is a scale factor, and *b* is the initial *θ* value.

### 2.6. Two-Step TRAP Assay

Telomerase activity was measured by the telomere repeat amplification protocol (TRAP) assay according to the protocol provided with the TRAPEZE telomerase detection kit (EMD Millipore). The two-step telomere repeat amplification protocol (tsTRAP) assay, an improved TRAP assay, is useful for reducing ligand effects on PCR amplification [40]. In the tsTRAP assay, the telomerase reaction mixture containing G-quadruplex-ligand is diluted and used as a template for the PCR step described below. Thus, diluted G-quadruplex-ligand did not affect PCR efficiency. Each 10-µL telomerase reaction mixture contained telomerase, 1 × TRAP reaction buffer, 1 × dNTP mix, and 0.2 µL TS primer, and either contained 2 µL of a defined concentration of G-quadruplex-ligand or lacked G-quadruplex-ligand. In the first step, each reaction was incubated at 30 °C for 60 min, then heated at 90 °C for 10 min. In the second step, telomerase reaction products were amplified in a 10-µL PCR reaction mixture containing 50-fold diluted telomerase mixture, 0.2 µL TS primer, 0.2 µL TRAP primer mix, 1 × dNTP mix, 1 × LA Taq polymerase buffer, and LA Taq polymerase (Takara Bio). PCR was carried out over 30 cycles of denaturation at 94 °C for 30 s, annealing at 59 °C for 30 s, and extension at 72 °C for 30 s. The TRAP assay products were resolved by non-denaturing electrophoresis at 400 V through a 10% nondenaturing polyacrylamide gel in Tris-borate-EDTA buffer (pH 8.5). The gels were stained with GelStar nucleic acid gel stain manufactured by Cambrex Corporation (East Rutherford, NJ, USA) and imaged using FLS-5100 film manufactured by Fuji Film Co., Ltd. (Tokyo, Japan). For the negative control reactions, lysis buffer was added instead of telomerase. For the positive control reactions, telomerase extract was added to a reaction solution that lacked any G-quadruplex-ligand. Relative activity (*A*) was calculated using the following equation:

(3) $$A=\{(X-X_{0}/C\}/\{(X_{p}-X_{0}/C_{p}\}$$

where X is the signal intensity of the region of the gel lane corresponding to the TRAP product ladder bands and C is the signal intensity of the region of the gel lane corresponding to the internal control product. Subscripts “*p*” and “*0*” indicate the positive and negative controls, respectively.

### 2.7. Fluorescence Intensity Analysis

GDDCs with FITC and Dabcyl at the 5' and the 3' ends, respectively, with various concentrations of rEGFR were heated at 80 °C for 2 min and gently cooled at 2 °C·min^−1^ to 20 °C in a buffer containing 50 mM MES-LiOH (pH 7.0), 100 mM KCl, and 10 mM MgCl_2_. Fluorescence intensity at 520 nm was measured at 25 °C using a fluorescence spectral scanning reader (Varioskan flash; Thermo Fisher Scientific Inc., Waltham, MA, USA) with excitation at 482 nm.

## 3. Results and Discussion

### 3.1. Design of GDDC

**Figure 2:**
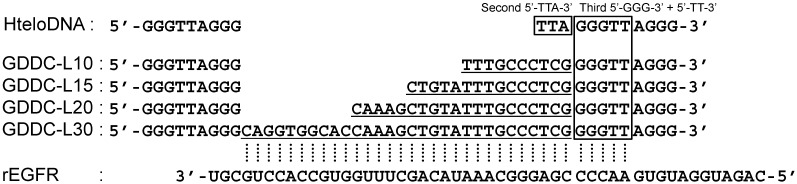
Sequences of HteloDNA and the GDDCs with various loop sequences complementary to rEGFR. The long loop region is underlined. Dashed lines represent the complementary relationship between GDDC and rEGFR.

EGFR mRNA is an important target mRNA for GDDCs used for cancer therapy because EGFR mRNA is highly transcribed in cancer cells [33]. Four GDDCs (GDDC-L10, L15, L20 and L30) were designed to target a specific sequence in EGFR mRNA (rEGFR), as shown in Figure 2. The GDDC sequences are based on a human telomeric DNA sequence (HteloDNA), 5'-GGGTTAGGGTTAGGGTTAGGG-3', because this oligo DNA forms a stable G-quadruplex structure in the presence of potassium ion [13,30,31,32]. The four GDDCs contain a 10-30-mer length loop in place of the second 5'-TTA-3' in HteloDNA; these loops are complementary to the target sequence in EGFR mRNA. Importantly, the target sequence contains 5'-AACCC-3' at the 5' end. It was expected that these five bases would hybridize with the third 5'-GGG-3' and the adjacent 5'-TT-3' of the GDDCs, leading to efficient unfolding of the GDDCs, since the 5'-GGG-3' region is involved in the formation of the G-quadruplex.

**Figure 3:**
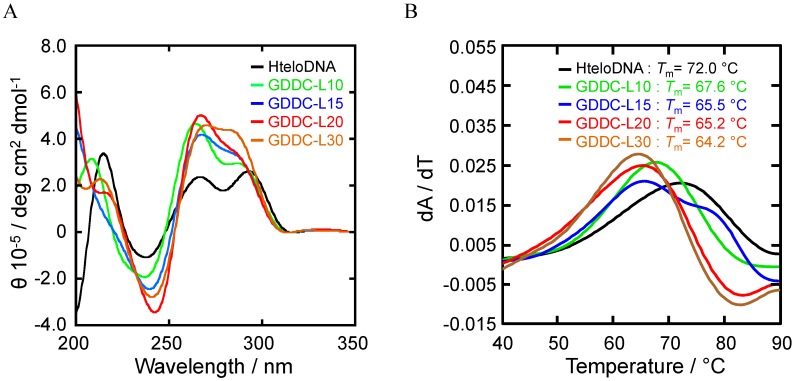
(**A**) CD spectra of HteloDNA and each GDDC at 25 °C in a buffer comprising 50 mM MES-LiOH (pH 7.0), 100 mM KCl, and 10 mM MgCl_2_. Before measurement, the DNA sample was heated at 80 °C for 2 min, then gently cooled at 2 °C·min^−1^; (**B**) dA/dT plots derived from UV melting curves of G-quadruplexes formed by HteloDNA and each GDDC in the same buffer as above were measured at 295 nm. Before measurement, the DNA samples were heated at 90 °C for 1 min, then gently cooled to 0 °C at 0.5 °C·min^−1^. The DNA samples were then heated from 0 to 90 °C at 0.5 °C·min^−1^.

To confirm that the GDDCs and HteloDNA form the G-quadruplex structure, CD spectra of the annealed GDDCs and HteloDNA were measured in a buffer containing 50 mM MES-LiOH (pH 7.0), 100 mM KCl, and 10 mM MgCl_2_ at 25 °C (Figure 3A). All the GDDCs and HteloDNA showed a CD spectrum with positive peaks at 260 and 295 nm and a negative peak at 240 nm, although there were subtle differences among peak intensities. The profiles indicate that all the GDDCs formed a (3 + 1) G-quadruplex structures comparable to HteloDNA and that the long loops did not significantly impact structure [34,35,36]. We further studied thermal stability of the G-quadruplexes. UV melting curves of the GDDCs and HteloDNA at 295 nm were analysed under the same conditions [40]. The melting temperature (*T*_m_) of HteloDNA was estimated to be 72.0 °C based on dA/dT plots derived from the hypochromic UV melting profile (Figure 3B). The *T*_m_ values of GDDC-L10, L15, L20 and L30 were 67.6, 65.5, 65.2 and 64.2 °C, respectively. Although these results indicate that progressively longer loops destabilized the G-quadruplex structure, it was confirmed that the G-quadruplex structures formed by the GDDCs were sufficiently stable for drug delivery around 37 °C.

### 3.2. Selection of G-Quadruplex Ligand for GDDC

**Figure 4:**
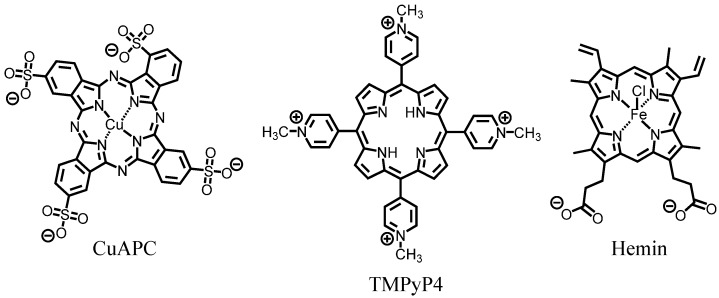
Chemical structures of CuAPC, TMPyP4, and hemin.

G-quadruplex ligand for the GDDC should show high specificity for the G-quadruplex structure over double-stranded DNA (dsDNA) because the ligand should be released from the unfolded GDDC containing dsDNA. In addition, the G-quadruplex ligand should efficiently inhibit telomerase to provide a high anticancer effect. Three representative G-quadruplex ligands (CuAPC, TMPyP4, and hemin; Figure 4), were screened to identify the most suitable ligand for the GDDC. 

**Figure 5:**
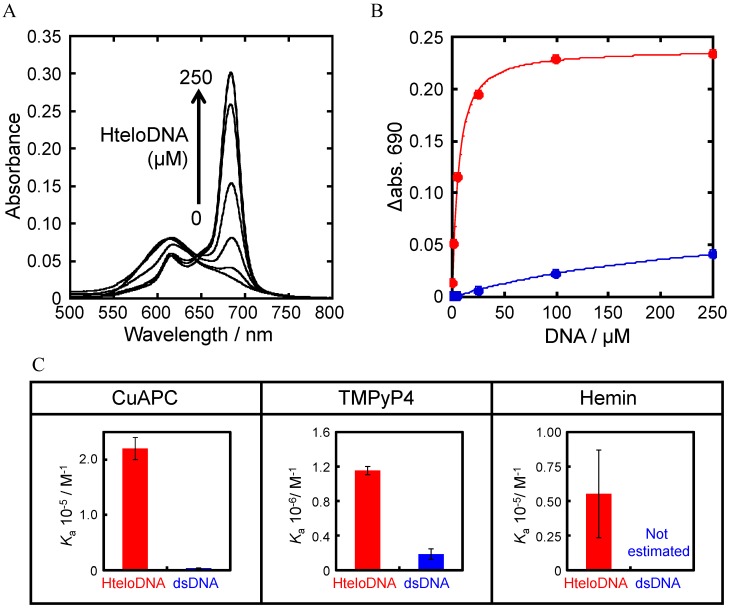
(**A**) Visible absorbance spectra of 2.5 µM CuAPC with 0–250 µM of HteloDNA at 25 °C. The sample was first heated at 80 °C for 2 min, then gently cooled at 2 °C·min^−1^; (**B**) Absorbance of 2.5 µM CuAPC at 690 nm with 0-250 µM of HteloDNA and dsDNA (5'-AGAAGAGAAAGA-3'/5'-TCTTTCTCTTCT-3'); (**C**) *K*_a_ value of each ligand for HteloDNA and dsDNA. All measurements were carried out in a buffer containing 50 mM MES-LiOH (pH 7.0), 100 mM KCl, and 10 mM MgCl_2_ at 25 °C.

The specificity of the ligands for the HteloDNA G-quadruplex was measured from absorbance spectra of the ligands with annealed HteloDNA in 50 mM MES-LiOH (pH 7.0), 100 mM KCl, and 10 mM MgCl_2_ buffer at 25 °C. The CuAPC spectrum showed a new peak around 690 nm that increased in intensity as the HteloDNA concentration increased (Figure 5A), indicating that CuAPC bound to the HteloDNA G-quadruplex, as reported previously [13,30,31,32]. The stoichiometry of the binding was studied by titration experiment with higher concentrations of CuAPC and HteloDNA (100 μM Cu-APC and 0–500 μM Htelo-DNA). A stoichiometric point around 100 μM Htelo-DNA was observed (Figure S1), suggesting a 1:1 binding of Cu-APC and Htelo-DNA. This result indicates that it is valid to use Equations (1) and (2) based on the single interaction (1:1 binding) model. The association constant (*K*_a_) was estimated to be (2.2 ± 0.2) × 10^5^ M^−1^ (*K*_d_ = 4.5 μM) at 25 °C (Figure 5B). In contrast, samples of CuAPC with the oligo dsDNA, 5'-AGAAGAGAAAGA-3'/5'-TCTTTCTCTTCT-3', showed a small increase in absorbance at 690 nm and the *K*_a_ value was (3.7 ± 0.4) × 10^3^ M^−1^ (*K*_d_ = 270.3 μM) at 25 °C (Figure 5B). These results demonstrate that CuAPC bound to the G-quadruplex with a two-order higher affinity than to the oligo dsDNA (Figure 5C). Similarly, the *K*_a_ value of TMPyP4 for the G-quadruplex was (1.2 ± 0.1) × 10^6^ M^−1^ (*K*_d_ = 0.8 μM) at 25 °C, which was 6-fold greater than that for the dsDNA, (1.8 ± 0.6) × 10^5^ M^−1^ (*K*_d_ = 5.6 μM) at 25 °C (Figure S2A and Figure 5C). The *K*_a_ value of hemin for the G-quadruplex was (5.5 ± 3.1) × 10^4^ M^−1^ (*K*_d_ = 18.2 μM) at 25 °C, but the change in the absorbance spectra of hemin with 0–250 μM dsDNA was too small to allow estimation of the *K*_a_ value (Figure S2B), indicating that hemin bound to the G-quadruplex with at least 14-fold greater affinity than to the oligo dsDNA (Figure 5C). Thus, CuAPC and hemin were more suitable for the GDDC than TMPyP4 because of their higher specificity for the G-quadruplex. 

**Figure 6:**
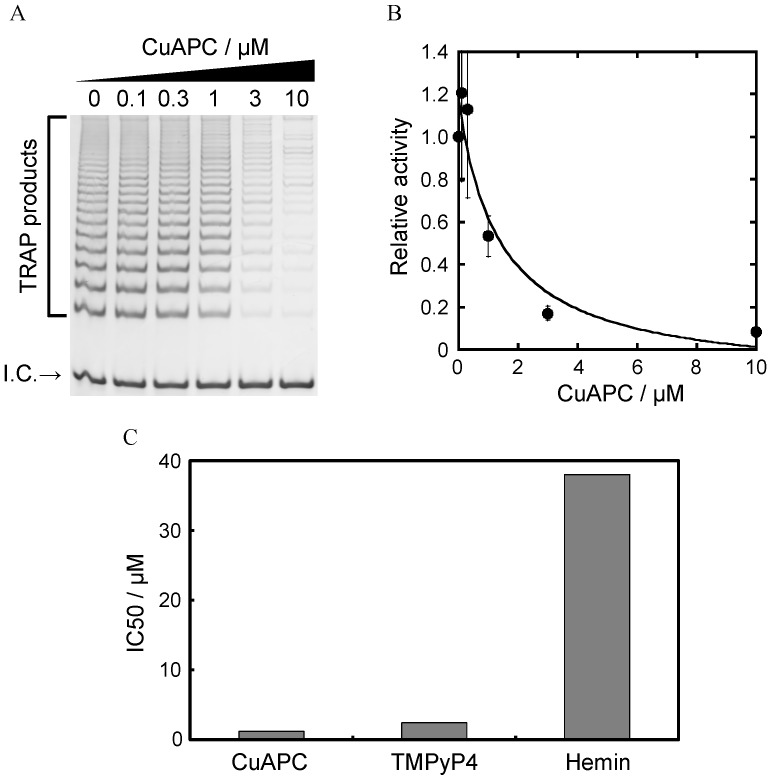
(**A**) Electrophoresis results from the two-step TRAP assay with 0–10 µM CuAPC. I.C. indicates the internal control for PCR amplification; (**B**) Relative activity of telomerase with 0–10 µM CuAPC. The relative activity value of 1 corresponds to the positive control (*i.e*., without CuAPC); (**C**) Comparison of the IC50 value of each ligand for telomerase inhibition.

Next, the telomerase inhibitory effect of the three ligands was studied using a two-step TRAP (tsTRAP) assay [41]. The telomerase reaction was conducted with HeLa cell lysate and each ligand, and then the 50-fold diluted product was amplified by PCR. The samples were diluted to limit the influence of each ligand on PCR. Higher concentrations of CuAPC reduced the amount of the TRAP assay product (Figure 6A). Telomerase can bind to the human telomeric DNA, 5'-(TTAGGG)_n_-3', via hybridization using its complementary RNA component with the human telomeric DNA, and add the same repetitive telomeric DNA sequence [42,43]. However, the G-quadruplex folded by the human telomeric DNA can not hybridize with the RNA component, which causes telomerase inhibition [42,43]. CuAPC can bind to the human telomeric G-quadruplex as demonstrated in the binding experiment with HteloDNA. Therefore, it is reasonable to assume that CuAPC can inhibit telomerase activity by binding to and stabilize the human telomeric G-quadruplex in telomerase reaction products [13,30,31,32]. The IC50 value (the concentration of ligand at 50% relative activity, see Experimental) was estimated to be 1.4 μM (Figure 6B), which is identical to the value previously reported [13,30,31,32]. Inhibition by hemin and TMPyP4 was also confirmed [13,30,31,32], with the IC50 values estimated to be 38 μM and 2.3 μM, respectively (Figures S3, S4 and Figure 6C). These results indicate that CuAPC and TMPyP4 were superior telomerase inhibitors to hemin. The high specificity of CuAPC to the G-quadruplex and its high telomerase inhibitory effect indicate that of the three ligands tested, CuAPC is best suited for use in a GDDC.

### 3.3. Affinity of CuAPC to GDDCs

**Figure 7:**
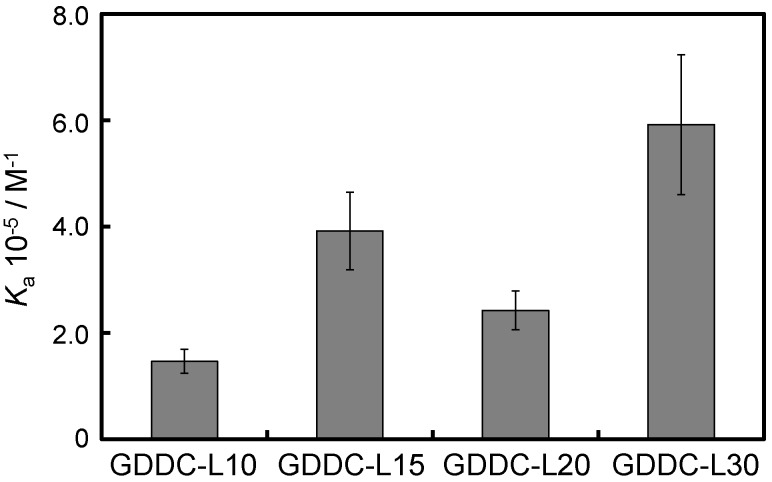
*K*_a_ values of CuAPC for GDDC with various loop lengths (10 to 30 bases). All measurements were carried out in 50 mM MES-LiOH (pH 7.0), 100 mM KCl, 10 mM MgCl_2_ buffer at 25 °C.

Based on the ligand binding experiments to the HteloDNA G-quadruplex and the telomerase inhibition experiments, CuAPC was selected as the drug for the GDDC. We further investigated the affinity of CuAPC for GDDCs by measuring the absorbance spectra of CuAPC with the annealed GDDCs. The same increase in the absorbance of CuAPC around 690 nm was observed upon the addition of GDDC-L10 as was observed with HteloDNA (Figures S5A and S5E). The *K*_a_ value was estimated to be (1.5 ± 0.2) × 10^5^ M^−1^ (*K*_d_ = 6.7 μM) at 25 °C (Figure 7), which is almost the same as that for HteloDNA. The new peak around 690 nm was also observed with other GDDCs (Figure S5B–E) and the *K*_a_ values for GDDC-L15, GDDC-L20, and GDDC-L30 were (3.9 ± 0.7) × 10^5^ M^−1^ (*K*_d_ = 2.6 μM), (2.4 ± 0.4) × 10^5^ M^−1^ (*K*_d_ = 4.2 μM), and (5.9 ± 1.3) × 10^5^ M^−1^ (*K*_d_ = 1.7 μM) at 25 °C, respectively (Figure 7). These results indicate that GDDC-L30 shows the highest affinity for CuAPC and is the most appropriate carrier for CuAPC among the four GDDCs tested. In addition, these results imply that longer loops tend to cause higher affinity with CuAPC except GDDC-L20, although longer loops destabilize the G-quadruplex structure (Figure 3B). Given that previous structural studies demonstrated that many G-quadruplex ligands bind to G-quadruplexes by end-stacking, longer loops of GDDC-L15 and GDDC-L30 may not stack on the G-quartet but may interact with CuAPC end-stacking on the G-quartet. The sandwich-like interaction of CuAPC between the longer loops and the G-quartet may enhance the affinity of GDDCs to CuAPC [44,45,46,47,48,49].

### 3.4. Unfolding of GDDCs by rEGFR

**Figure 8:**
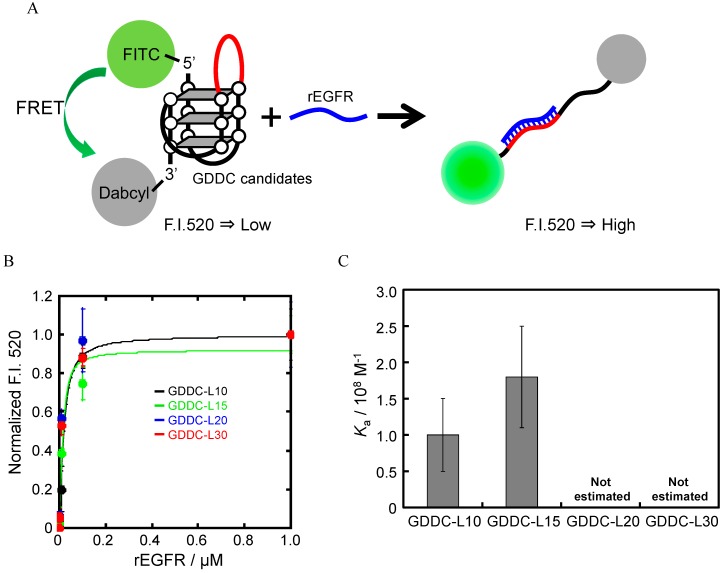
(**A**) Strategy to study the unfolding of GDDCs by Regfr; (**B**) Normalized fluorescence intensity (F.I.) of each GDDC (20 nM) at 520 nm with 0–10 µM rEGFR. (B) *K*_a_ values of rEGFR for HteloDNA and each GDDC. All measurements were carried out in a buffer containing 50 mM MES-LiOH (pH 7.0), 100 mM KCl, and 10 mM MgCl_2_ at 25 °C. *K*_a_ values of GDDC-L20 and GDDC-L30 were too high to evaluate accurately.

The GDDC must undergo a structural transition in response to the target mRNA in order to be useful as a DDS. The unfolding of the GDDC G-quadruplexes by the target sequence in EGFR mRNA was studied by modifying the GDDCs with a fluorescent probe (FITC) and a quencher probe (Dabcyl) at the 5' end and the 3' end, respectively (Figure 8A) [50]. Double-labeled GDDCs folded into the G-quadruplex structure position the FITC and Dabcyl probes close enough for fluorescence quenching, whereas their distance in the unfolded G-quadruplex should be large enough to allow significant FITC fluorescence. As expected, the fluorescence intensity of GDDC-L10 at 520 nm increased with increasing rEGFR concentration, mimicking an increase in the concentration of EGFR mRNA (Figure 8B). This result indicates that the G-quadruplex structure formed by GDDC-L10 unfolded due to hybridization between the 10-mer length loop and rEGFR. The unfolding of GDDC-L15, L20, and L30 in response to rEGFR was similarly demonstrated (Figure 8B). The *K*_a_ values for GDDC-L10 and L15 were estimated to be (1.0 ± 0.5) × 10^8^ M^−1^ (*K*_d_ = 0.01 μM) and (1.8 ± 0.7) × 10^8^ M^−1^ (*K*_d_ = 0.006 μM) at 25 °C, respectively (Figure 8C). However, the *K*_a_ values for GDDC-L20 and L30 could not be calculated because of their very high affinity for rEGFR. These results were as predicated because GDDC-L20 and L30 can form a longer duplex with rEGFR than GDDC-L10 and L15. Therefore, the G-quadruplex structure of GDDC-L30 should unfold at lower concentrations of EGFR mRNA than GDDC-L10, L15, and L20. The concentration of each mRNA in cells is extremely low (pM level). Thus, GDDC-L30, which is expected to bind to the mRNA of EGFR with the highest affinity among GDDCs, should be best suited as the drug delivery carrier stimulated by the mRNA.

### 3.5. Release of CuAPC from GDDC-L30 in Response to rEGFR

**Figure 9:**
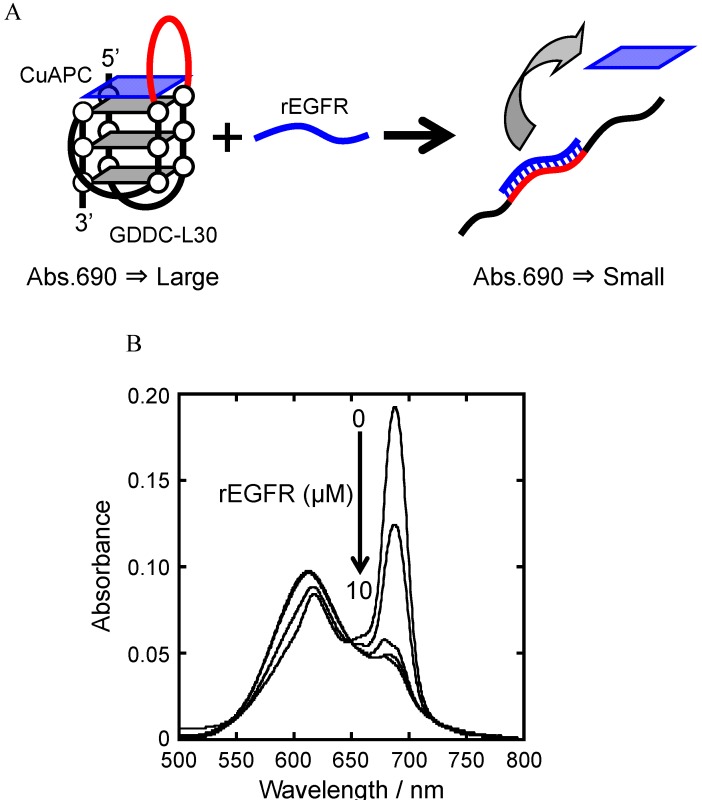
(**A**) Strategy to study the release of CuAPC from GDDC-L30 in response to rEGFR; (**B**) Visible absorbance spectra of 2.5 µM CuAPC/2.5 µM GDDC-L30 complex with 0–10 µM rEGFR. The sample was heated at 80 °C for 2 min, the gently cooled at 2 °C·min^−1^ before measurement.

The results showed that CuAPC is an appropriate drug for GDDCs because of its high specificity to the G-quadruplex and high telomerase inhibitory effect, and that GDDC-L30 is an appropriate carrier because of its high affinity for CuAPC and rEGFR. The release of CuAPC from GDDC-L30 in response to rEGFR was determined by measuring the absorbance spectra of CuAPC in the presence of increasing concentrations of rEGFR (Figure 9A). CuAPC annealed with GDDC-L30 in the absence of rEGFR provided a large peak around 690 nm (Figure 9B). As described above, this large peak demonstrates binding of CuAPC to GDDC-L30. The intensity of the peak decreased as the concentration of rEGFR increased (Figure 9B), indicating that CuAPC was released from GDDC-L30 into free solution in response to rEGFR. Furthermore, the results obtained with GDDCs modified with FITC and Dabcyl suggest that the 30-mer length loop of GDDC-L30 hybridizes with rEGFR, causing unfolding of the G-quadruplex and release of CuAPC from GDDC-L30. Taken together, our results suggest that the proposed DDS using GDDC can release the anticancer drug CuAPC in response to EGFR which is overexpressed in cancer cells specifically.

## 4. Conclusions

We studied the properties of four GDDCs differing in loop length (10–30 mer) as drug delivery carriers, and screened three G-quadruplex ligands (CuAPC, TMPyP4, and hemin) as suitable drugs. Each GDDC candidate formed a sufficiently stable (3 + 1) G-quadruplex structure for DDS. More importantly, each GDDC candidate bound CuAPC, a specific G-quadruplex ligand with telomerase inhibitory effect. The G-quadruplex structure unfolded by binding with rEGFR. Furthermore, we demonstrated that GDDC-L30 could release CuAPC in response to rEGFR. These results indicate that this GDDC should be useful for anticancer therapy with few side-effects. The mRNA of EGFR exists in a single cell at pM level [51]. Since the *K*_d_ value of GDDC-L30 to rEGFR is also pM level, the mRNA of EGFR is expected to unfold GDDC-L30 in cells. However, the concentration of CuAPC released from the unfolded GDDC-L30 is not enough for telomerase inhibition, because its IC50 value for the telomerase inhibition is 1.4 μM. Thus, the G-quadruplex ligand with higher telomerase inhibitory effect is required for practical application of GDDC. Although further improvements of ligand inhibitory function are out of scope of this study, there are other specific G-quadruplex ligands with high telomerase inhibitory effect such as telomestatin, phthalocyanine derivatives [19,21,22]. These G-quadruplex ligands may be useful for improvements of GDDC *in vivo*.

## Supplementary Files

Supplementary File 1
