# Supplementary material for: A mRNA-Responsive G-Quadruplex-Based Drug Release System
Source: Sensors (Basel). 2015 Apr 21;15(4):9388–403. doi: 10.3390/s150409388 (PMC4431183; doi:10.3390/s150409388)
Supplement: Supplementary File 1 [file sensors-15-09388-s001.pdf]

OPEN ACCESS

sensors

ISSN 1424-8220
www.mdpi.com/journal/sensors

Supplementary Information

A mRNA-Responsive G-Quadruplex-Based Drug Release System. *Sensors* 2015, *15*, 9388-9403

Hidenobu Yaku [1], Takashi Murashima [2], Daisuke Miyoshi [2,*] and Naoki Sugimoto [2,3]

1. Advanced Research Division, Panasonic Corporation, 3-4 Hikaridai, Seika-cho, Soraku-gun, Kyoto 619-0237, Japan; E-Mail: yaku.hidenobu@jp.panasonic.com
2. Faculty of Frontiers of Innovative Research in Science and Technology (FIRST), Konan University, 7-1-20 Minatojima-Minamimachi, Chuo-ku, Kobe 650-0047, Japan; E-Mails: murasima@konan-u.ac.jp (T.M.); sugimoto@konan-u.ac.jp (N.S.)
3. Frontier Institute for Biomolecular Engineering Research (FIBER), Konan University, 7-1-20 Minatojima-Minamimachi, Chuo-ku, Kobe 650-0047, Japan

* Author to whom correspondence should be addressed; E-Mail: miyoshi@center.konan-u.ac.jp; Tel: +81-78-303-1426; Fax: +81-78-303-1495.

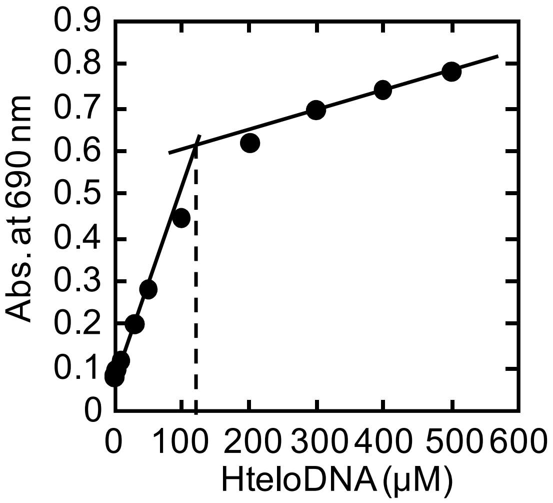

Figure S1. Change in absorbance at 690 nm of 100 μM Cu-APC with 0–500 μM Htelo-DNA in a buffer containing 50 mM MES-LiOH (pH 7.0), 100 mM KCl, and 10 mM $MgCl_2$ at 25 °C.

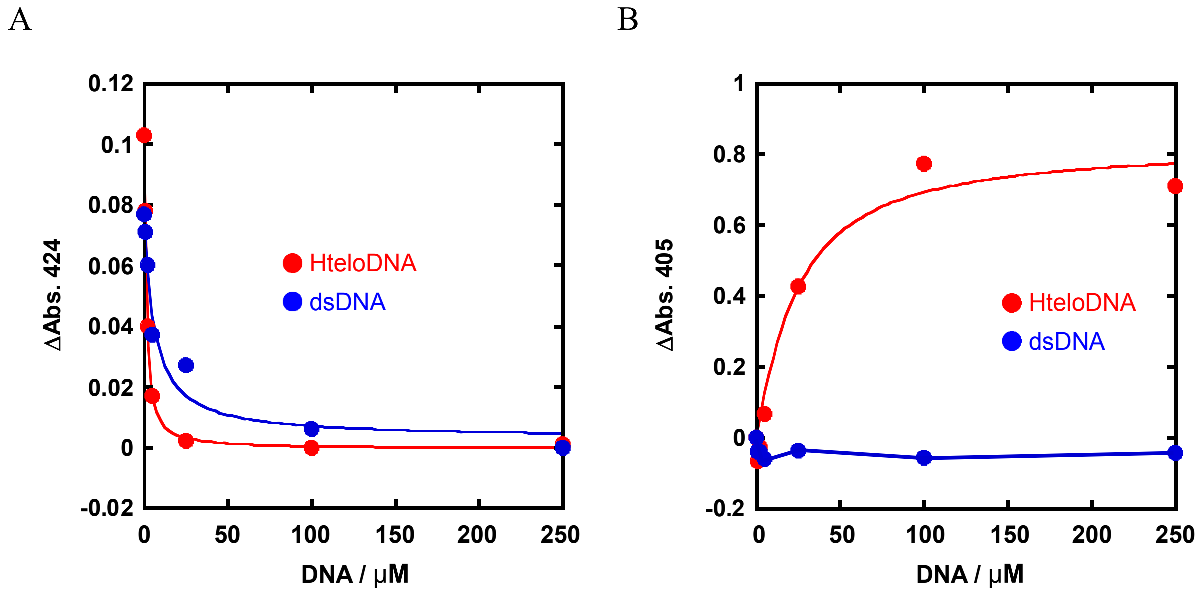

Figure S2. (**A**) Absorbance of 1.0 μM TMPyP4 at 424 nm (A) and 12.5 μM Hemin at 405 nm (**B**) with 0-250 μM of HteloDNA and 0–250 μM of dsDNA (5'-AGAAGAGAAAGA-3'/5'-TCTTTCTCTTCT-3'). Before measurement, the sample was heated at 80 °C for 2 min, gently cooled at 2 $°C·min^{-1}$. All measurements were carried out in a buffer containing 50 mM MES-LiOH (pH 7.0), 100 mM KCl, and 10 mM $MgCl_2$ at 25 °C.

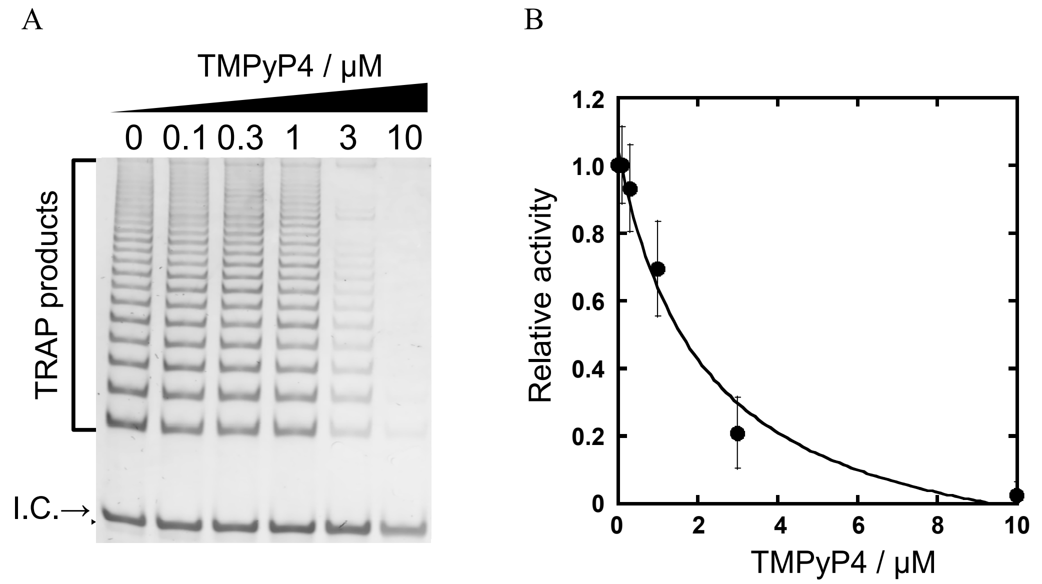

Figure S3. (**A**) Electrophoresis result of the two-step TRAP assay with 0–10 μM of TMPyP4. I.C. indicates the internal control for PCR amplification; (**B**) Relative activity of telomerase with 0–10 μM of TMPyP4. The relative activity value of 1 corresponds to the positive control, namely without TMPyP4.

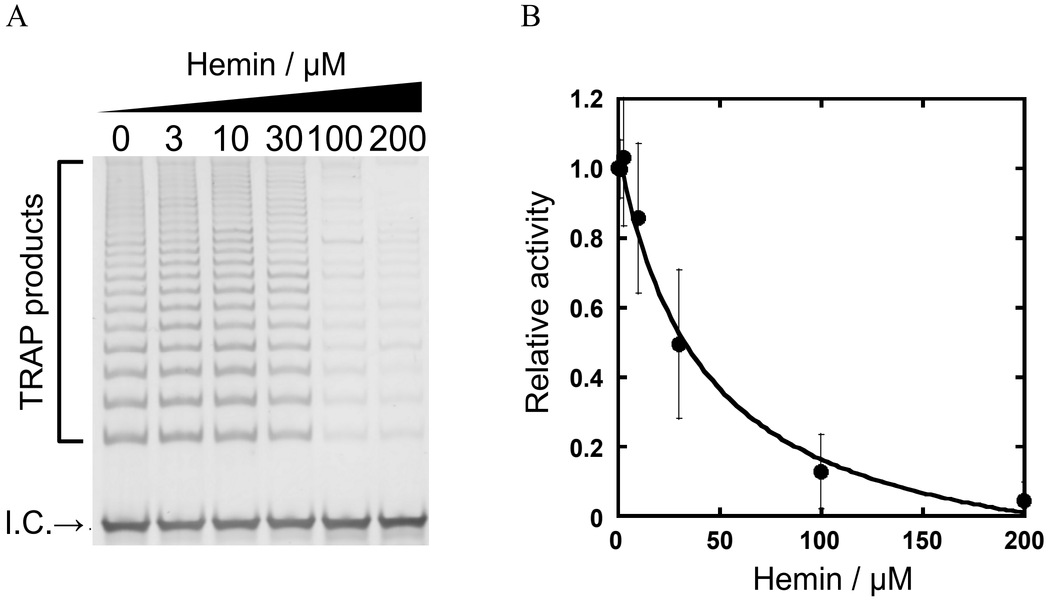

Figure S4. (**A**) Electrophoresis result of the two-step TRAP assay with 0–200 μM of Hemin. I.C. indicates the internal control for PCR amplification; (**B**) Relative activity of telomerase with 0–200 μM of Hemin. The relative activity value of 1 corresponds to the positive control, namely without Hemin.

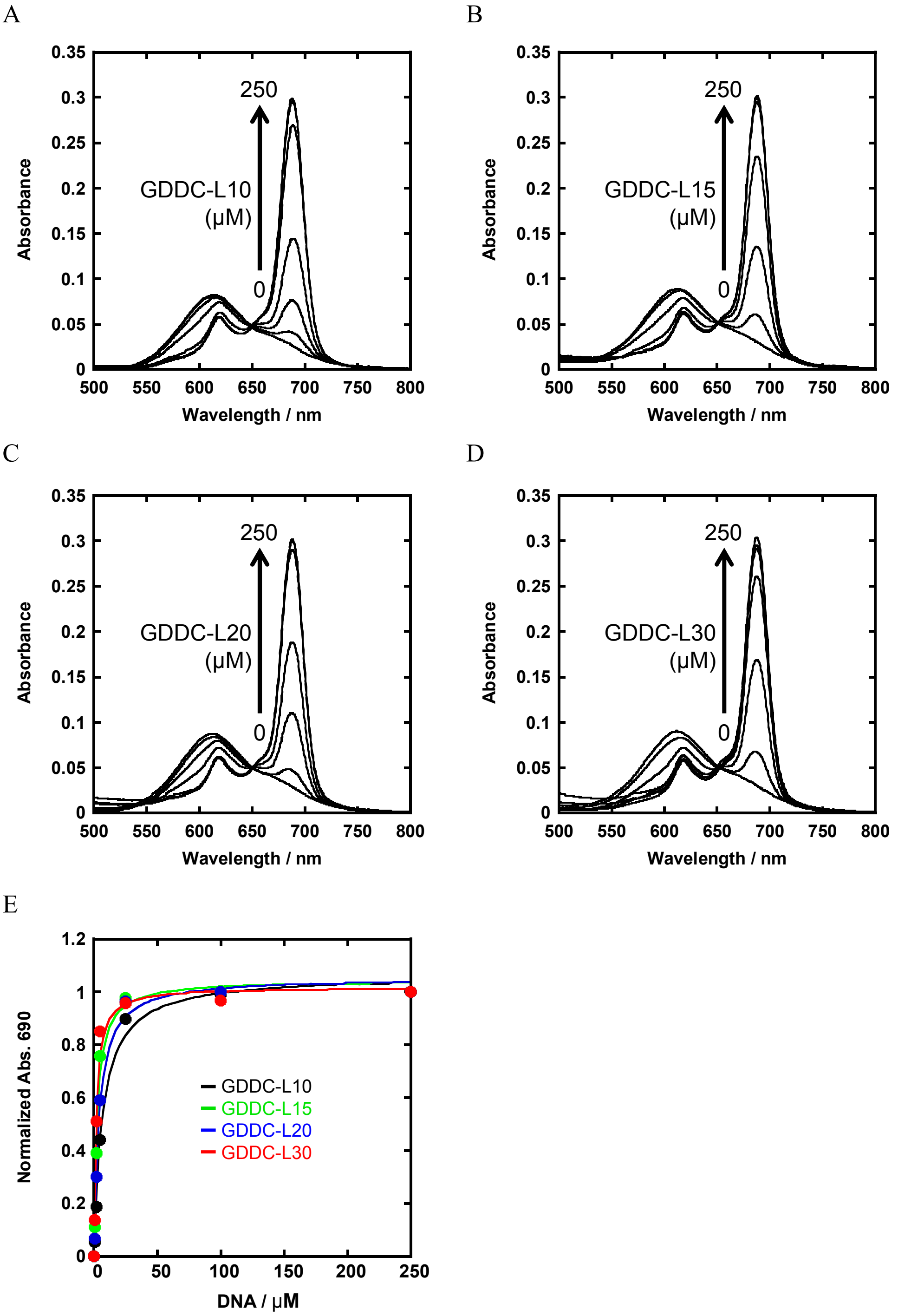

Figure S5. Visible absorbance spectra of 2.5 µM CuAPC with 0–250 µM of GDDC-L10 (**A**); GDDC-L15 (**B**); GDDC-L20 (**C**); and GDDC-L30 (**D**). Before measurement, the samples were heated at 80 °C for 2 min, gently cooled at 2 °C·min^{-1}; (**E**) Normalized absorbance of 2.5 µM CuAPC at 690 nm with 0–250 µM of each GDDC candidate. All measurements were carried out in a buffer containing 50 mM MES-LiOH (pH 7.0), 100 mM KCl, and 10 mM $MgCl_2$ at 25 °C.

© 2015 by the authors; licensee MDPI, Basel, Switzerland. This article is an open access article distributed under the terms and conditions of the Creative Commons Attribution license (http://creativecommons.org/licenses/by/4.0/).
